# Land use diversity and park use in New York City

**DOI:** 10.1016/j.pmedr.2021.101321

**Published:** 2021-02-04

**Authors:** Dustin Fry, J. Aaron Hipp, Claudia Alberico, Jing-Huei Huang, Gina S. Lovasi, Myron F. Floyd

**Affiliations:** a Drexel University Dornsife School of Public Health, Department of Epidemiology and Biostatistics, 3600 Market Street 7^th^ Floor, Philadelphia, PA 19104, USA; b North Carolina State University College of Natural Sciences, Department of Parks, Recreation, and Tourism Management, 5112 Jordan Hall, 2800 Faucette Drive, Raleigh, NC 27695, USA

**Keywords:** Park use, Land-use diversity, Physical activity, Soparc, Built environment

## Abstract

Neighborhood parks and mixed-use land development are both understood to be important independent contributors to physical activity levels. It has been hypothesized that mixed-use land development could increase park use as a result of mixed-use neighborhoods being consistently activated throughout the day, but the results of previous research on this question have been inconsistent and the mediational role of neighborhood activation has not been tested. This study leverages data from Google Places Popular Times and the National Establishment Time Series to directly test the mediational role of the daily temporal distribution of neighborhood activation, to construct a novel measure of commercial activity diversity, and to help disentangle built-environment density from commercial diversity. Park use data was measured from 10,004 systematic observations of 20 neighborhood parks in New York City in the spring and summer of 2017. The hypothesis that commercial activity diversity is positively associated with park use was not supported in any models. However, a positive relationship between built-environment density and park use was found, which may help to explain prior inconsistent findings.

## 1 Introduction

Neighborhood parks and mixed-use land development are both considered to be important determinants of walking and physical activity. Parks can provide a safe, attractive, and no-cost setting for adults and children to exercise and socialize (Bedimo-Rung et al., 2005) and can contribute meaningfully to physical activity levels (Han et al., 2013). Likewise, when neighborhoods provide a large variety of amenities in a small area (e.g., residential, office, retail/commercial, and public space), residents are conveniently close to a variety of useful destinations (Saelens et al., 2008); research suggests that this can promote the decision to walk for transportation, thereby increasing physical activity levels (Saelens et al., 2003, Christian et al., 2011).

These two independent causes of physical activity may also act cooperatively through a process of “neighborhood activation,” a condition in which the businesses and public spaces of a neighborhood are populated and in use. Jacobs (1961) hypothesized that because different types of businesses operate at different times of the day, areas with a single type of commercial activity tend to be activated during only certain hours and are abandoned for the rest of the day. In contrast, areas with high commercial activity diversity are activated consistently throughout the day as different businesses are patronized and as their employees come and go. As a result, parks in areas of high commercial activity diversity will experience incidental use throughout the day as they suit the convenience of people who are in the neighborhood primarily for reasons other than visiting a park. This creates a consistent level of “eyes on the street,” also known as natural surveillance (Foster and Giles-Corti, 2008), that increases the perceived safety of the park and in turn encourages a greater level of use from people who would not feel comfortable entering an otherwise deserted park. The hypothesis predicts that commercial activity diversity will be positively associated with park use at all times of the day, a relationship that is mediated by the consistency of the temporal distribution of daily neighborhood activation (Jacobs, 1961).

The relationship between mixed-use land development and park use has been assessed in prior research with mixed results. In a study across Boston, Cincinnati, and San Diego, one study found that land use mix measured as *perceived walkable access* to a variety of destinations was positively associated with children’s park use and physical activity, but that land use mix measured as *perceived land use diversity* was not (Rosenberg et al., 2009). Another study found a positive relationship between an index of land use mix and active park use among older adults in Bogotá (Parra et al., 2010). In an analysis from the current study, Huang et al. found a positive relationship between an index of land use mix and children’s park use in New York City (Huang et al., 2020). Conversely, however, a study of a mid-sized Canadian city found that greater land use diversity was associated with *lower* odds of a park being used for physical activity, the opposite direction as expected (Kaczynski et al., 2010).

A better understanding of the relationship between land use diversity and park use, and a test of the mediating role of the temporal patterns of neighborhood activation, may require more nuanced measures of the built environment. Indices of land use mix such as those used by Parra et al., 2010, Kaczynski et al., 2010, Huang et al., 2020 are based on the relative ratios of residential space, commercial space, and office space surrounding parks. Although the ratio of all commercial land use to residential land use is a meaningful measure of neighborhood context, it does not on its own provide information about the type or variety of commercial activities present. A neighborhood where the majority of the commercial activity consists of nightclubs and convenience stores will be qualitatively different than one where the commercial activity is a more comprehensive mix of (for example) restaurants, shops, medical providers, museums, and gyms, but these two neighborhoods may be measured the same by a land use mix index if the total amount of commercial space and residential space is the same, as may be the case in comparing two dense mixed-use neighborhoods within a large urban city. This limitation inhibits a comprehensive examination of the relationship between the built environment and park use. In contrast, perceived measures of land use diversity such as were used by Rosenberg et al. (2009) may be more appropriate in capturing aspects of neighborhood context relevant for park use, but may also be vulnerable to human biases and recall error.

The present study builds on previous work and seeks to more directly assess how mixed-use land development, temporal activation of neighborhoods, and park use may be associated in low-income minority neighborhoods in New York City. To do this, we leverage Popular Times data from Google Places and business data from the National Establishment Time Series. Together, these data sources can be used to derive novel measures of neighborhood context that may be more relevant to addressing the hypothesis that the diversity of commercial activities in a neighborhood is positively associated with park use, mediated by the temporal distribution of neighborhood activation.

## 2 Methods

### 2.1 PARC^3^ park use data

Between May and August of 2017, a sample of 20 parks in New York City was assessed using the SOPARC protocol (Huang et al., 2020, Marquet et al., 2019a, Marquet et al., 2019b), a validated direct-observation instrument for assessing park use and park-based physical activity (McKenzie et al., 2006). Parks were selected based on being within low-income census tracts with a high proportion of children and with populations that were predominantly Latino or Asian. Within each sampled park, target areas were identified based on features such as playground sets, courts, and fields that are likely to support children’s physical activity; parks in the current study ranged from 4 to 13 target areas. During two to three hour-long observation periods on each day that a park was assessed, each target area was visited in sequence to complete a “round.” At each target area, the population within the target area was “scanned” to approximate an instantaneous measure of the number of people in the target area according to gender, age, race, and level of physical activity. These observations consisted only of observations of public behavior, and therefore met criteria for a human subjects exemption.

Each park was assessed on two weekdays and on each weekend day during the spring (May-June) and again during the summer (July-August) for a total of 8 visits per park. During the spring, parks were assessed at 3:00P.M., 4:30P.M., and 6:00P.M. while in the summer observation periods took place at 10:00 A.M. and at 6:00P.M. Four rounds per hour were attempted, but not all rounds were completed for all observation periods, with a minimum of 1 completed round. Overall, 162 visits were completed, including 375 observation periods and 10,003 scans of target areas, with 4,513 scans being completed by two or more scorers simultaneously. An estimated total of 50,879 park users were observed overall.

### 2.2 CPAT park quality

To control for aspects of park quality that may be relevant for park use, the Community Park Audit Tool (CPAT) was used (Kaczynski et al., 2012). CPAT assesses aspects of park quality through six composite variables: park access amenities, safety concern present in parks, safety or appearance concerns present in surrounding neighborhood within sight on any side of the park, the number of usable facilities, the number of usable amenities, and the number of aesthetic features. Each domain was standardized to 100, and each park was assigned an overall score based on the mean across all six domains.

### 2.3 Google Places Popular times

Google Places is a large online database of information on “points of interest,” including commercial businesses, transit stations, government buildings, and landmarks (Places | Google Maps Platform. Google Cloud. Accessed November 25, 2019); in this paper, these points of interest are referred to as “establishments.” Data on individual establishments is downloadable through the Google Places API.

**Fig. 1:**
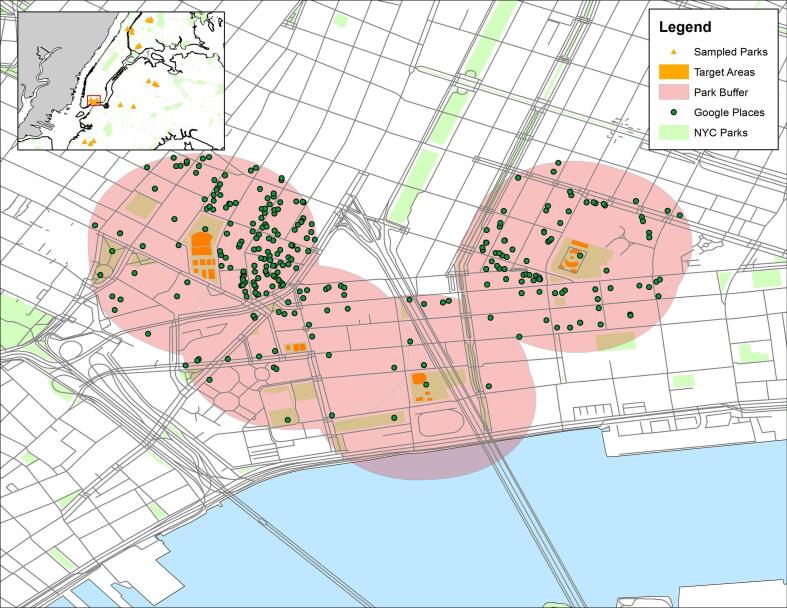
Buffers and downloaded Google Places establishments for four sampled parks in the Lower East Side of Manhattan, NYC. All sampled parks are displayed in the inset map. Water area and road network data from Open Street Maps.

To obtain data on the neighborhood context surrounding parks, 250-meter buffers were constructed around the target areas of each park. Buffers were drawn around target areas rather than park boundaries because the full park was not assessed for some large parks. Neighborhood amenities closer to the assessed target areas are more likely to be relevant for their use than are neighborhood amenities closer to other areas of the park. Within these buffers, all establishments with popular times data were downloaded using the populartimes Python library (m-wrzr. Populartimes, 2019) between October 31st and November 2nd, 2018, as illustrated in Fig. 1. A total of 672 unique establishments were identified.

The Popular Times data from Google Places includes a value for each hour for each day in the week, representing the relative level of use of the establishment at that hour (Popular times, wait times, and visit duration - Google My Business Help, 2019). The popularity value ranges from 0 to 100, with 0 representing that the establishment is closed at that time, and 100 representing the hour during the week with the highest level of use of that establishment. The data also include the location of the establishment and a list of categories to describe it, such as “restaurant,” “hardware store,” or “beauty salon.”

Google Places data were leveraged in three ways: to measure land use density, the diversity of commercial activity, and the consistency of the temporal distribution of neighborhood activation. Land use density was measured as the total number of establishments within each park buffer. This measure serves as a statistical control to ensure comparability across diverse neighborhoods and may also be an independent predictor of park use.

To measure the diversity of commercial activity, each establishment was initially categorized based on the first category in its list. Because many of these 54 unique categories appeared to be qualitatively similar, the raw categories were grouped into 15 broader categories to aid in interpretation. For example, “clothing store,” “shoe store” and “department store” were all grouped together within “Retail/services” (see Supplementary Table 1). These broader categories are hereafter referred to as “types,” and the number of types of establishments within each park buffer was used as a measure of commercial activity diversity.

Finally, the Popular Times data were used to create a measure of the consistency of the temporal distribution of neighborhood activation within each buffer during the average day. This measure is the L^2^ (Euclidean) norm of the daily distribution of popularity in each park’s buffer. To do this, the mean daily popularity vector for each establishment was calculated by taking the mean popularity of each hour across the week. In Equation (1), this mean daily popularity vector is represented by **pop**, with each individual establishment represented as *i* out of *n* total establishments in each park’s buffer. For each park, for every hour *h*, the popularity value for each establishment *i* is summed and divided by the total summed popularity values for every establishment in the entire 24-hour day. This ratio represents the estimated conditional probability that a visitor to any establishment in a park’s buffer on an average day will be there at *h* hour. This ratio is squared, each hour is summed, and the square root of the sum taken. This value can range between $\frac{1}{\sqrt{24}}=0.204$, representing a perfectly uniform distribution of popularity across the average day in a park’s buffer, and 1, representing a situation in which all establishments in a park’s buffer are simultaneously and exclusively patronized during a single hour of the day.

(1) Lpark2=∑h=023∑i=1npopi,h∑h=023∑i=1npopi,h2

Because the number of types of establishments increases as neighborhood commercial activity diversity increases, our hypothesis suggests a positive relationship between the number of types of establishments and park use. Conversely, a larger L^2^ norm corresponds with a less-even temporal distribution of daily neighborhood activation, so we predict a negative relationship between L^2^ norm and park use.

Although our hypothesis assumes that an even temporal distribution of neighborhood activation will increase park use at all times of the day, this may not be true. A sensitivity analysis was conducted in which the L^2^ norm was only computed between the 9am hour and the 7 pm hour (one hour before the earliest park observation and one hour after). The results of this analysis are presented in Supplementary Table 3.

### 2.4 National establishment Time Series data

One important limitation is that only a small percentage of all establishments in the Google Places database have Popular Times data (approximately 6%, based on a subsample in which all establishments were downloaded instead of only establishments with Popular Times data). As a secondary analysis, data from the National Establishment Time Series (NETS) for 2014 were used. These data provide geocoded information for businesses as well as non-profit organizations and government offices, and provide an effectively exhaustive listing of all establishments in the United States (Neumark et al., 2005). Each of the 24,395 unique establishments within the park buffers was supplemented with an imputed daily popularity vector: NETS establishments were recategorized into the same categories as the Google Places establishments (see Supplementary Table 2) and assigned the popularity vector of the nearest Google Places establishment of the same category. The same measures (number of places, commercial diversity, and L^2^ norm) were computed from the NETS data.

Although the NETS data may provide a more valid measure of commercial activity diversity, the imputed popularity vectors may not be a better measure of the daily distribution of neighborhood activation. At the level of the establishment in the Google Places data, daily popularity varies within as well as between categories, so imputed popularity vectors may not accurately reflect the popularity distribution for a given NETS establishment. However, we assume that the strengths and weaknesses inherent to this approach are complementary to the main analysis.

### 2.5 Statistical analysis

The two park-level measures of neighborhood commercial activity diversity and the temporal distribution of neighborhood activation were analyzed in Poisson generalized estimating equations (GEE). Because of the varying sizes of parks and differing number of target areas, as well as the inconsistent number of rounds completed, each model used as its outcome the total number of people counted in each scan of a target area. This measure is comparable across parks of different sizes. Target areas were clustered within parks and each model controlled for the total number of establishments within each buffer as a measure of neighborhood density as well as the CPAT park quality score. The total number of establishments was Z-score standardized to ensure comparable effect sizes between the Google Places analysis and the NETS analysis. The number of unique establishment types within each buffer and the L^2^ norm were analyzed in separate models and together to assess their effect on park use. Because of the hypothesized mediating effect of the L^2^ norm on the relationship between the number of types of establishments and park use, the hypothesis predicts (1) a positive relationship between the number of types of establishments and park use; (2) a negative relationship between the L^2^ norm and park use; and (3) when both are in the same model, the effect size of the number of types of establishments will be attenuated.

Separate models were computed based on the Google Places data and for the NETS secondary analysis. All statistical analysis was conducted in R version 3.5.1 (R Core Team, 2018) and generalized estimating equations were computed using the GEE package (Vincent et al., 2019).

## 3 Results

Table 1, Table 2 present descriptive statistics for each park, with the neighborhood context measures presented separately as computed from the Google Places data or from the NETS data. Comparing these two sets of measures, the number of establishments is significantly correlated (r = 0.909, p < 0.001) as is the number of types of establishments in each buffer (r = 0.549, p = 0.012) but the L^2^ norm computed directly from the Google Places data is not significantly correlated with the L^2^ norm computed from the imputed NETS data (r = −0.016, p = 0.944). The much-larger number of establishments within each buffer computed from NETS data reflects its greater comprehensiveness above the set of Google Places establishments with popular times data.

**Table 1:** Park and neighborhood context measures.

					Google Places		NETS	
Park	Area (m^2^)	Target Areas (n)	CPAT	Visitors (SD)	n Estab.	n Types	n Estab.	n Types
Hart Playground	3,832	7	52.15	4.79 (5.44)	18.00	9.00	949	13
Nelson Playground	4,871	11	48.52	1.86 (3.18)	19.00	10.00	604	12
Brizzi Playground	2,987	6	59.24	6.91 (5.34)	21.00	7.00	894	13
Frank D O'Connor Playground	6,479	13	54.62	6.60 (8.05)	34.00	12.00	1,266	13
Maria Hernandez Park	28,181	12	51.65	7.91 (7.38)	62.00	8.00	1,123	11
Rappaport Playground	4,833	9	52.70	3.56 (5.25)	15.00	5.00	817	12
Seward Park	13,466	9	64.51	4.91 (4.72)	84.00	12.00	3,669	14
Coleman Square Playground	10,514	4	49.75	2.98 (6.91)	14.00	6.00	1,362	13
Moore Homestead Playground	8,052	11	53.25	7.39 (5.14)	48.00	12.00	1,426	14
People's Park	5,772	13	51.99	2.34 (3.21)	16.00	9.00	481	13
Playground One	1,854	4	52.81	3.42 (4.37)	63.00	10.00	3,753	14
Saint Mary's Park	144,626	9	61.85	5.39 (4.67)	12.00	5.00	380	12
Slattery Playground	3,799	10	59.55	3.73 (5.65)	26.00	9.00	875	12
Sternberg Park	16,433	6	51.64	7.42 (9.30)	31.00	10.00	1,209	14
Columbus Park	12,576	11	55.69	7.28 (5.98)	164.00	11.00	5,363	13
Rainbow Playground	2,001	7	48.32	11.86 (11.49)	23.00	9.00	990	13
Washington Park	2,153	5	49.20	2.89 (4.47)	17.00	7.00	624	12
Webster Playground	3,145	8	46.18	3.60 (4.49)	45.00	9.00	743	14
Highbridge Park	311,052	4	65.43	4.88 (6.38)	8.00	6.00	524	13
Merriam Playground	3,338	8	52.95	3.04 (4.39)	8.00	6.00	343	12

Park area, number of target areas, CPAT park quality score, average number of visitors per scan, and the number of establishments and number of types of establishments in each buffer as computed from Google Places and NETS data. Parks are ordered based on the L^2^ norm as computed directly from Google Places data (Table 2).

**Table 2:** Daily distributions of neighborhood activation and L^2^ Norm.

	Google Places		NETS Imputed Data	
Park	Average Daily Distribution	L^2^	Average Daily Distribution	L^2^
Hart Playground		0.23		0.22
Nelson Playground		0.24		0.22
Brizzi Playground		0.25		0.30
Frank D O'Connor Playground		0.25		0.26
Maria Hernandez Park		0.25		0.26
Rappaport Playground		0.25		0.31
Seward Park		0.25		0.26
Coleman Square Playground		0.26		0.28
Moore Homestead Playground		0.26		0.26
People's Park		0.26		0.28
Playground One		0.26		0.27
Saint Mary's Park		0.26		0.24
Slattery Playground		0.26		0.28
Sternberg Park		0.26		0.26
Columbus Park		0.27		0.28
Rainbow Playground		0.27		0.30
Washington Park		0.27		0.28
Webster Playground		0.27		0.28
Highbridge Park		0.28		0.22
Merriam Playground		0.29		0.21

Average daily distribution of neighborhood activation and L^2^ norm for each park as computed directly from Google Places data and from NETS imputed data. Parks are ordered based on the L^2^ norm as computed directly from Google Places.

Table 3 presents results from modeling. Using measures computed from Google Places data, there is no significant association between the number of types of establishments in a park’s buffer and park use, controlling for the total number of places and the park quality score. There is also no significant relationship between the L^2^ norm of the distribution of daily popularity in each park’s buffer, controlling for the total number of establishments in the park’s buffer and the park quality score. When both the number of types of places in the park’s buffer and the L^2^ norm are together in the same model, neither is significant. These results are consistent using measures computed from the NETS data.

**Table 3:** Modeling Results.

		*n* Types		L^2^ Norm		*n* Establishments		Park Quality	
Data	Model	*b*	95% CI	*b*	95% CI	*b*	95% CI	*b*	95% CI
Google Places	*n* Types	0.029	(-0.043, 0.101)			0.102	(−0.007, 0.212)	0.004	(−0.034, 0.041)
	L2 Norm			1.073	(−14.405, 16.552)	0.128	(0.035, 0.221)*	0.003	(−0.033, 0.039)
	*n* Types + L2 Norm	0.035	(−0.052, 0.122)	2.860	(−14.629, 20.348)	0.089	(−0.064, 0.242)	0.004	(−0.033, 0.040)
NETS	n Types	0.053	(−0.213, 0.319)			0.099	(−0.001, 0.199)	0.001	(−0.035, 0.037)
	L2 Norm			3.329	(−4.411, 11.070)	0.096	(0.004, 0.188)*	0.004	(−0.030, 0.038)
	*n* Types + L2 Norm	0.044	(−0.224, 0.312)	3.224	(−4.212, 10.659)	0.084	(−0.031, 0.199)	0.004	(−0.030, 0.038)

Modeling results are presented separately as computed directly from Google Places data and from NETS imputed data. The number of places in each buffer is Z-score standardized.

*p < 0.05

However, there is an apparent positive relationship between land use density and park use. In the model predicting park use from the number of types of places and park use, there is a nonsignificant positive trend between the total number of establishments in the buffer and park use. In the model predicting park use from the L^2^ norm, there is a significant positive relationship between the number of establishments and park use. This result is also consistent between the Google Places and NETS analyses. While not supporting the main hypothesis of the analysis, this finding provides an alternate and policy-relevant explanation for between-neighborhood differences in park use.

### 3.1 Comparison with land use mix

The null relationship between commercial activity diversity and park use found in this analysis is apparently contradictory to the positive relationship between land use mix and park use found in Huang et al.’s analysis of the same park use data. (Huang et al., 2020) However, this difference may be explained by the two different measures used. The land use mix index used in the Huang et al. analysis is not correlated with the number of types of establishments surrounding each park in the sample (Google Places: r = 0.32, p = 0.174; NETS: r = 0.37, p = 0.104) but is positively correlated with the number of establishments in each buffer (Google Places: r = 0.60, p = 0.004; NETS: r = 0.63, p = 0.003). This suggests that, in at least this context, a land use mix index may be a more direct measure of land-use density, particularly the density of commercial uses, than of the variety of commercial land uses present.

## 4 Discussion

Taken together, the primary analysis using Google Places data directly and the secondary analysis using NETS data with an imputed daily popularity vector fail to support the hypothesis that parks surrounded with a greater level of commercial activity diversity experience greater levels of use, as mediated by the evenness of the temporal distribution of daily neighborhood activation. However, the positive relationship between the number of establishments in each park’s buffer and park use may be important.

Comparing this result with previous work highlights the need to disentangle measures of land use density from land use diversity in order to better understand the relationship between the built environment and park use. Our approach may be most analogous to Rosenberg et al. (2009). Their measure of perceived walkable access to a variety of destinations may approximate a measure of density, so their finding that *access* predicted park use while *diversity* did not is a complimentary result to this analysis, and similarly suggests the importance of disentangling closely-related neighborhood measures. As larger-scale data become available, researchers should take advantage of the opportunity to construct measures of the neighborhood environment that encode theory-relevant information in a more direct way than existing land-use indices. For example, the Built Environment and Health-Neighborhood Walkability Index leverages NETS data to incorporate information about “pedestrian trip generating/supporting establishments” in a measure of neighborhood walkability (Rundle et al., 2019). If “big data” is guided by theory-based frameworks to address specific hypotheses, a more complete and actionable understanding of how the built environment influences health can be approached.

Several limitations to the current study should be noted. First, only a small fraction of neighborhood establishments have Popular Times data, and these may not be a representative sample. As a result, our measurement of the temporal distribution of daily neighborhood activation may not be valid, either as calculated directly from the Google Places data or through the imputed NETS dataset. Notably, no schools within the park buffers had popular times data, although large nearby schools may be important drivers of park use in several parks in the sample. Second, the target areas selected for this study were tailored to assess children’s physical activity, and so do not include certain features such as walkways that may be disproportionately used by adults visiting the park incidentally. Finally, the NETS data corresponds with a time period three years prior to the park observations, while the Google Places data was gathered one year after the observations. The density and mix of business types are unlikely to have changed quickly enough to substantively impact our findings, but any change would tend to exaggerate the discordance between the NETS and Google Places metrics.

By leveraging a novel data source in Google Places Popular Times data, this study seeks to gain a more complete understanding of the relationship between neighborhood context and park use. Park visits are influenced not only by characteristics of the park itself, but also by the area surrounding it. Policymakers seeking to promote physical activity may wish to locate parks in densely developed areas where they are most likely to be used, to preserve parkland in neighborhoods that are densifying, and to prioritize programmatic activities in parks in denser urban neighborhoods. Future research can evaluate how neighborhood-level built environment interventions can increase park use and physical activity levels.

## CRediT authorship contribution statement

**Dustin Fry:** Conceptualization, Methodology, Investigation, Data curation, Formal analysis, Visualization, Writing - original draft. **J. Aaron Hipp:** Methodology, Investigation, Writing - review & editing, Funding acquisition, Project administration, Supervision. **Claudia Alberico:** Methodology, Investigation, Data curation, Writing - review & editing. **Jing-Huei Huang:** Methodology, Investigation, Writing - review & editing. **Gina S. Lovasi:** Methodology, Investigation, Writing - review & editing, Supervision. **Myron F. Floyd:** Methodology, Investigation, Writing - review & editing, Funding acquisition, Project administration, Supervision.

## Declaration of Competing Interest

The authors declare that they have no known competing financial interests or personal relationships that could have appeared to influence the work reported in this paper.
