# Supplementary material for: Land use diversity and park use in New York City
Source: Prev Med Rep. 2021 Feb 4;22:101321. doi: 10.1016/j.pmedr.2021.101321 (PMC9366970; doi:10.1016/j.pmedr.2021.101321)
Supplement: Supplementary data 1 [file mmc1.docx]

Supplemental Table 1

| **Establishment Type (Recoded)** | **Google Places Category** |
| --- | --- |
| Automotive | - car_repair |
|  | - gas_station |
|  | - car_wash |
|  | - car_dealer |
|  | - car_rental |
|  | - parking |
| Bar/nightclub/liquor | - bar |
|  | - night_club |
|  | - liquor_store |
| Convenience/pharmacy | - pharmacy |
|  | - convenience_store |
| Entertainment/arts/museum | - art_gallery |
|  | - museum |
|  | - movie_theater |
| Financial/legal/government | - bank |
|  | - atm |
|  | - finance |
|  | - courthouse |
|  | - local_government_office |
|  | - accounting |
|  | - insurance_agency |
|  | - lawyer |
|  | - police |
| Grocery | - grocery_or_supermarket |
|  | - bakery |
|  | - supermarket |
| Healthcare | - doctor |
|  | - health |
|  | - dentist |
|  | - hospital |
|  | - veterinary_care |
|  | - physiotherapist |
| Other | - point_of_interest |
|  | - travel_agency |
|  | - route |
|  | - neighborhood |
|  | - premise |
|  | - storage |
| Park/gym | - park |
|  | - gym |
| Post office/library | - post_office |
|  | - library |
| Religious | - church |
|  | - synagogue |
|  | - mosque |
|  | - place_of_worship |
| Restaurant/cafe | - restaurant |
|  | - cafe |
|  | - meal_delivery |
|  | - meal_takeaway |
| Retail/services | - store |
|  | - clothing_store |
|  | - shoe_store |
|  | - electronics_store |
|  | - laundry |
|  | - jewelry_store |
|  | - furniture_store |
|  | - home_goods_store |
|  | - hardware_store |
|  | - book_store |
|  | - shopping_mall |
|  | - department_store |
|  | - pet_store |
|  | - florist |
|  | - bicycle_store |
| Salon/spa | - beauty_salon |
|  | - hair_care |
|  | - spa |
| Transit | - subway_station |
|  | - bus_station |
|  | - transit_station |

Supplemental Table 2

| **Establishment Type (recoded)** | **NETS Category** |
| --- | --- |
| Automotive | - Gas stations – SIC code based definition |
| Bar/nightclub/liquor | - Bars and nightclubs serving alcohol |
|  | - Liquor stores |
|  | - Nightclubs not serving alcohol |
|  | - Strip Clubs/Gentlemen’s Clubs |
|  | - Other Social clubs |
| Convenience/pharmacy | - Convenience Store/Gas stations - Chain name based definition |
|  | - Convenience stores - Chain name based definition |
|  | - Convenience stores – SIC code based definition |
|  | - Pharmacies/Drug Stores – SIC Code based definition |
|  | - Pharmacies/Drug Stores – Chain name based definition |
| Entertainment/arts/museum | - Amusement parks, carnival, rodeo |
|  | - Arcades |
|  | - Gambling |
|  | - Museum and art gallery |
|  | - Performance based entertainment |
|  | - Non-Physical Activity Recreation Clubs |
|  | - Professional/Semi-professional sports and stadium entertainment |
|  | - Zoo, aquarium, arboretum |
| Financial/legal/government | - Banks |
|  | - Credit unions |
|  | - Fire services and stations |
|  | - Political Organizations |
|  | - Police services and stations |
|  | - Individual and family social services |
| Grocery | - Small grocers/bodegas |
|  | - Fish markets |
|  | - Fruit and vegetable markets |
|  | - Medium-sized grocers |
|  | - Meat and dairy (animal fats) |
|  | - Health food, natural food, vitamins |
|  | - Other food stores |
|  | - Supermarkets – SIC code based definition |
|  | - Supermarkets - Chain name based definition |
| Healthcare | - Behavioral Health Hospitals |
|  | - Behavioral Health Outpatient and Continuous Care |
|  | - Dental Care |
|  | - Hospitals and major medical centers |
|  | - Offices or clinics of health practitioners |
|  | - Kidney Centers |
|  | - Mental Health Hospitals |
|  | - Mental Health Outpatient and Continuous Care |
|  | - Physical Therapy |
|  | - Residential facilities with health care |
|  | - Retail clinics |
|  | - Urgent care |
| Other | - Day care |
|  | - Junior, Community, and Technical Colleges |
|  | - Preschool |
|  | - Elementary and secondary schools |
|  | - Tourist |
|  | - Colleges/University |
|  | - Not categorized |
| Park/gym | - Child-based Light/moderate physical activity |
|  | - Child-based Multi-use facilities |
|  | - Child-based Vigorous physical activity |
|  | - Light/moderate physical activity |
|  | - Multi-use facilities |
|  | - Vigorous physical activity |
| Post office/library | - Libraries |
|  | - U.S. Postal Service |
| Religious | - Religious institutions |
| Restaurant/cafe | - Bakery, Candy, Ice Cream (sweets) – Chain name based definition |
|  | - Bakery, Candy, Ice Cream (sweets) – SIC code based definition |
|  | - Coffee shops - Chain name based definition |
|  | - Coffee shops – SIC Code Based definition |
|  | - Casual Dining - Chain name based definition |
|  | - Ethnic Restaurants – Other Asian |
|  | - Ethnic Restaurants – Popular Asian |
|  | - Other restaurants/eating places |
|  | - Ethnic Restaurants – Popular Ethnic |
|  | - Ethnic Restaurants – European |
|  | - Fast food Fast Casual - Chain name based definition |
|  | - Fast food – SIC code based definition |
|  | - Nuts |
|  | - Pizza |
|  | - Fast food Quick Service - Chain name based definition |
| Retail/services | - Conventional Mass Merchandise – Chain name based definition |
|  | - Discount Department Stores |
|  | - Department Stores |
|  | - General Mass Merchandise – Chain name based definition |
|  | - Laundromat/Dry cleaning |
|  | - Other Walkable Retail |
|  | - Supercenters - Chain name based definition |
|  | - Wholesale/Warehouse Club Stores – Chain name based definition |
|  | - Wholesale/Warehouse Club Stores – SIC code based definition |
| Salon/spa | - Beauty shops and barbers |
|  | - Massage parlors |
|  | - Spas |
|  | - Tanning Salons |

**Table 3: Sensitivity Analysis Modeling Results**

|  |  | ***n* Types** | | **L^2^ Norm** | | ***n* Establishments** | | **Park Quality** | |
| --- | --- | --- | --- | --- | --- | --- | --- | --- | --- |
| **Data** | **Model** | *b* | 95% CI | *b* | 95% CI | *b* | 95% CI | *b* | 95% CI |
| Google Places | *n* Types | 0.287 | (-0.044, 0.101) |  |  | 0.102 | (-0.008, 0.212) | 0.004 | (-0.034, 0.041) |
|  | L2 Norm |  |  | -5.141 | (-73.651, 63.368) | 0.132 | (-0.011, 0.295) | 0.003 | (-0.033, 0.039) |
|  | *n* Types + L2 Norm | 0.029 | (-0.042, 0.101) | 1.250 | (-70.262, 72.763) | 0.099 | (-0.088, 0.286) | 0.005 | (-0.028, 0.038) |
| NETS | n Types | 0.053 | (-0.213, 0.319) |  |  | 0.099 | (-0.001, 0.199) | 0.001 | (-0.035, 0.037) |
|  | L2 Norm |  |  | 17.832 | (-23.383, 59.048) | 0.076 | (-0.037, 0.189) | 0.004 | (-0.028, 0.038) |
|  | *n* Types + L2 Norm | 0.064 | (0.251, 0.380) | 18.744 | (-22.908, 60.395) | 0.056 | (-0.096, 0.207) | 0.005 | (-0.028, 0.038) |

Modeling results are presented separately as computed directly from Google Places data and from NETS imputed data. The number of establishments in each buffer is Z-score standardized. The L^2^ norm is computed only between the 9am hour and the 7pm hour.
